# Inducible limb-shaking transitory ischemic attacks: a video-documented case report and review of the literature

**DOI:** 10.1186/s12883-016-0601-8

**Published:** 2016-05-23

**Authors:** Sverre Rosenbaum, Christian Ovesen, Nancy Futrell, Derk W. Krieger

**Affiliations:** Department of Neurology, University of Copenhagen, Bispebjerg Hospital, Bispebjerg Bakke 23, Copenhagen, DK-2400 NV Denmark; Intermountain Stroke Center, 5292 College Dr 204, Salt Lake City, UT 84123 USA; Department of Neurology, University of Copenhagen, Rigshospitalet, Blegdamsvej 9, Copenhagen, DK-2100 Denmark

**Keywords:** Stroke, EC-IC bypass, Carotid occlusion, Limb-shaking TIA

## Abstract

**Background:**

Limb-shaking transient ischemic attack (TIA) is a well-recognized, but rare observation in contralateral carotid steno-occlusive disease. Consequently, most clinicians have not had the chance to witness an attack.

**Case presentation:**

We present the story of a 64-year old gentleman with exercise-induced weakness associated with tremor in his right arm. His left internal carotid artery was occluded at the bifurcation. Administration of statin and antiplatelet did not relieve his symptoms, and his stereotypic, exercise-induced “limb-shaking” episodes persisted. He underwent successful extracranial to intracranial (EC-IC) bypass, which stopped his symptoms. The patient, however, returned to our department and reported that he was able to recreate his original symptoms by compressing the bypass graft manually.

**Conclusion:**

To our knowledge, this is the first case with video documentation of the clinical appearance of a limb-shaking TIA. We hope this case report will increase the physicians’ understanding of the clinical nature of limb-shaking TIAs.

**Electronic supplementary material:**

The online version of this article (doi:10.1186/s12883-016-0601-8) contains supplementary material, which is available to authorized users.

## Background

Limb-shaking transient ischemic attack (TIA) is a well recognized, but rarely documented sequelae of contralateral carotid occlusion or severe stenosis [1, 2]. We report on limb-shaking TIAs in a patient with extracranial ipsilateral carotid occlusion alleviated by surgical revascularization (EC-IC bypass), which were later reproduced by the patient intentionally compressing his bypass graft. Video documentation demonstrating the ischemia induced movement disorder is available online (see Additional file 1).

## Case presentation

**Fig. 1:**
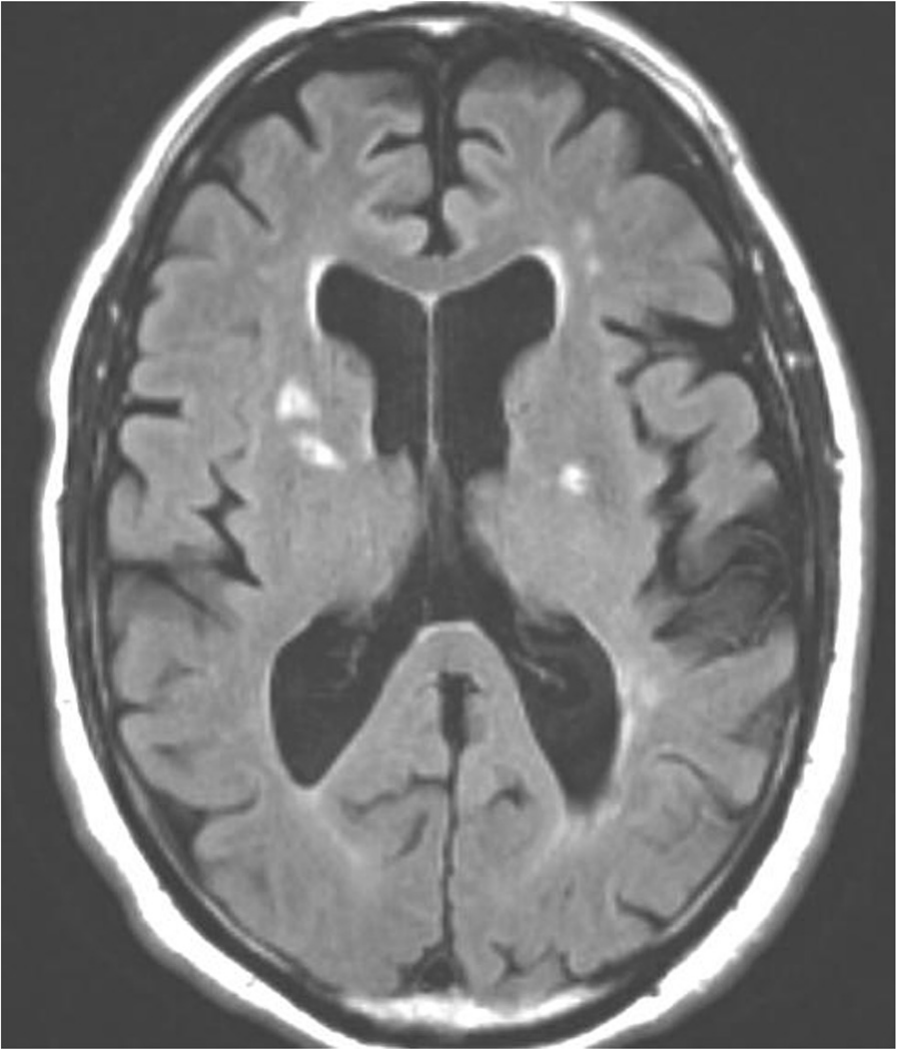
Initial MRI FLAIR sequence. MRI FLAIR sequence showing several bilateral areas of discreet cerebral ischemia. The diffusion weighted images were negative, suggesting an absence of acute cerebral infarction related to the continued limb-shaking TIAs

**Fig. 2:**
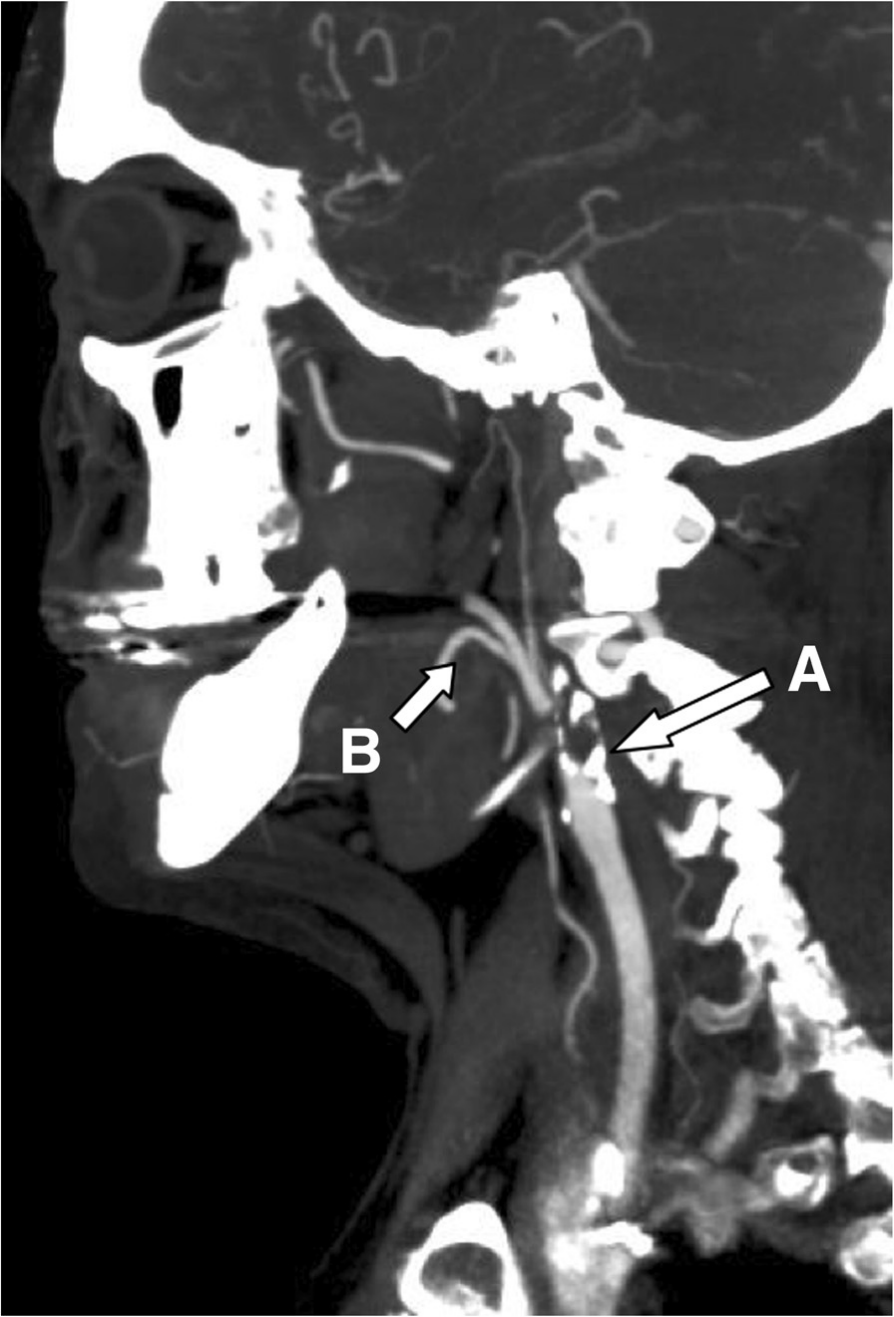
Initial CT-angiography showing left carotid occlusion. CT-angiography of the left carotid artery demonstrates calcified plaque of the left carotid bulb with atherosclerotic occlusion of the internal carotid artery (**a**). Of note the left external carotid artery is patent and there is robust filling of its branches (**b**)

A 64-year old man with known arterial hypertension, hypercholesterolemia, a 40 pack-year history of smoking and alcohol abuse, developed recurrent exercise-induced weakness associated with high-frequency irregular tremor in his right arm. In the beginning, the patient only experienced the symptoms during running/brisk walking. The patient later progressed to experience the symptoms during light walking. These shaking spells lasted for several minutes, but without other associated neurological deficits. MRI of the brain revealed no signs of acute ischemia in the left hemisphere, but bilateral subcortical white matter lesions, suggestive of small vessel disease, were present (Fig. 1). The left internal carotid artery (ICA) was occluded at the bifurcation (Fig. 2), but there were no additional stenoses or occlusions in the remainder of the cervico-cranial vasculature. The left middle cerebral artery (MCA) was reperfused from the circle of Willis due to anterior cross filling. On first admission, he was on no antihypertensive medication and had a blood pressure of 135/91 and a heart rate of 91.

**Fig. 3:**
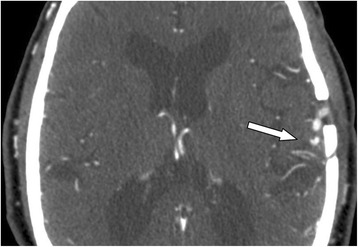
CT-angiography demonstrating the patency of the by-pass graft. CT-angiography after EC-IC bypass shows left craniotomy defect with pronounced distal MCA flow from the left superior temporal artery (*arrow*)

**Fig. 4:**
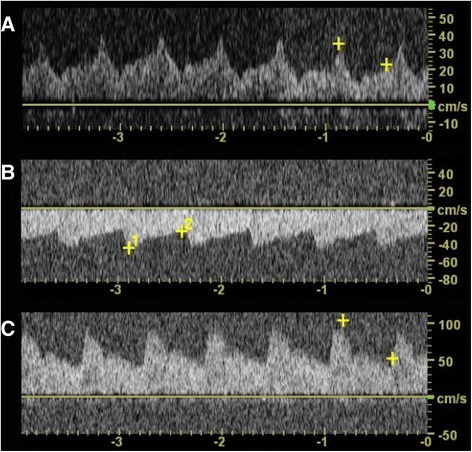
Transcranial doppler flow patterns. Sequential transcranial doppler (TCD) flow patterns are displayed. The left MCA is imaged via temporal bone window at a depth of approximately 5 cm. Panel **a** shows anterograde flow through the left MCA with the patient manually occluding the bypass graft (flow pattern *above* the line). The significance of the wave form is uncertain. Panel **b** display the retrograde flow pattern in the left MCA delivered through a patent EC-IC bypass graft (flow pattern *below* the line). In panel **c** is TCD done over the contralateral MCA showing a normal flow pattern

Administration of a statin and antiplatelet therapy did not ameliorate his symptoms, and he continued to have stereotyped ’limb-shaking’ TIAs precipitated by mild exertion. He underwent successful high-flow bypass between his superficial temporal artery and middle cerebral artery - extracranial to intracranial (EC-IC) bypass, with prompt cessation of the spontaneous ’limb-shaking’ TIA episodes (Fig. 3). Duplex Transcranial Doppler confirmed the patency of the bypass graft as demonstrated by reversal of flow direction in the left MCA (Fig. 4).

The patient was asymptomatic for a little over two years, but returned as an outpatient reporting that he could manually recreate his original symptoms by compressing the superficial temporal artery anastomosis (STA) just above the burrhole over the zygomatic bone. On examination, ‘limb-shaking’ could be provoked 20 s after compression and lasting for 20 s after releasing the pressure (see Additional file 1). The patient had electroencephalography (EEG) recording during an event, which was negative for epileptic discharges and focal slowing.

## Discussion

Historically, most TIAs have been attributed to cerebral embolism from small emboli with early recanalization [3]. There are, however, two types of TIAs. One of these are clearly caused by episodic hypoperfusion; limb-shaking TIAs [4, 5] and light-induced monocular vision loss [6]. These TIA syndromes are both associated with occlusion or severe stenosis of the carotid artery [7]. Limb-shaking TIAs generally occur in patients with carotid occlusive disease and exhausted cerebrovascular reserve [4]. A symptomatic event may be precipitated by decreased cerebral perfusion pressure, which can result from antihypertensive medications [8] or orthostatic hypotension [9]. It has also been described as a consequence of decreased cerebral perfusion resulting from shifts of blood flow within vascular beds, such as those induced by exercise [7, 9] or by the postprandial state [10]. Hyperventilation, which causes blood flow shifts intracranially, has been reported to cause limb-shaking TIAs in patients with Moya-Moya disease [11]. In our reported case, mild exercise induced the symptoms, presumably from increased skeletal muscle perfusion at the expense of cerebral perfusion.

Treatment of repetitive limb-shaking TIAs with standard preventative measures including platelet inhibition and statins was not effective in our patient or in cases reported in the literature [8]. Although no convincing evidence exists on the topic, successful surgical reperfusion in patients with repetitive limb-shaking TIAs, either with carotid endarterectomy or EC-IC bypass [2, 12], has been reported to successfully alleviate symptoms in the majority of the reported cases, as it did in this case.

The Carotid Occlusion Surgery Study (COSS) [13] compared medical stroke preventive therapy to EC-IC bypass grafting in patients with carotid occlusion based on both clinical and imaging criteria (increased oxygen extraction fraction). However, the study was halted due to futility. Although there was evidence of improved oxygen metabolism with EC-IC bypass, the surgical treatment provided no protection from stroke over medical therapy. This was mainly due to a high perioperative risk. In our reported case, medical therapy failed to stop symptoms, but surgical therapy was successful. The amelioration of distressing TIA symptoms was certainly interpreted as a success of the surgical procedure, but does not prove a stroke was prevented in our case.

It has been suggested that the surgery actually decreased the effectiveness of the ‘pre-surgical collateral’ flow to the left MCA in this patient. Prior to the surgery, limb-shaking TIAs occurred only with exercise, but not at rest. Two years following the surgery, during which time the collateral flow reserve was not essential, the patient was able to reproduce the TIA symptoms at rest by manually compressing the graft. This suggests that the effectiveness of the pre-existing collaterals decreased with establishment of the STA-MCA bypass impairing the recruitment blood flow through collaterals, when the by-pass grafts blood flow was disrupted by brief mechanical compression. This reduction of collateral flow is a theoretical risk of late complications of EC-IC bypass in the setting of traumatic disruption of the bypass graft.

This case also reinforces the concept that a movement disorder can be a manifestation of cerebral ischemia. The concept that “positive” symptoms are seizure and “negative” symptoms are stroke is a generalization that can result in diagnostic errors with both stroke and seizure patients [14, 15]. Hemiballismus and hemichorea are consequences of prior cerebral ischemia, which may become less frequent or entirely resolved over time [16]. Limb-shaking TIAs can be mistaken for seizures, if not carefully evaluated [14]. Importantly, in the reported patient, a separate episode was not accompanied by EEG changes (data not shown).

From the video of the patient’s episode, the onset from vascular compression to occlusion was approximately 20 s, and there was a further 20 s delay from reperfusion to the recovery from the symptoms. This observation is consistent with early experimental studies of cerebral ischemia [17–19]. Although the patient’s induced ischemic episodes have been short, and his episodes prior to his revascularization surgery did not produce permanent symptoms, the patient has been advised against compressing his STA.

A patient population of 313 patients with symptomatic occlusion of the internal carotid artery was found to have 11 % (34 patients) incidence of limb-shaking TIAs [7]. This suggests this particular TIA type may be more common than generally realized. TIAs are almost never seen by a physician, leaving the diagnosis to be made by history. To our knowledge, this is the first case with video documentation of the clinical appearance of a limb-shaking TIA, which should increase physician understanding of the clinical nature of limb-shaking TIAs.

## Conclusion

Limb-shakes are potential manifestations of acute stroke, but can easily be misdiagnosed as seizure activity. In addition, data does not support seizure activity as the pathophysiological background behind this phenomenon. Physicians need to keep this in mind when evaluating acute neurological patients.

## Additional file

Additional file 1: Video presentation of patient with Limb-shakin TIA. (M4V 16499 kb)

## Abbreviations

COSS: carotid occlusion surgery study
EC-IC bypass: extracranial to intracranial bypass
EEG: electroencephalography
ICA: internal carotid artery
MCA: middle cerebral artery
STA: superficial temporal artery anastomosis
TIA: transient ischemic attack
